# HIV and Sexual Dysfunction in Men

**DOI:** 10.3390/jcm10051088

**Published:** 2021-03-05

**Authors:** Sara De Vincentis, Giulia Tartaro, Vincenzo Rochira, Daniele Santi

**Affiliations:** 1 Unit of Endocrinology, Department of Biomedical, Metabolic and Neural Sciences, University of Modena and Reggio Emilia, Via Giardini 1355, 41126 Modena, Italy; sara.devincentis@unimore.it (S.D.V.); giuliatartaro@gmail.com (G.T.); daniele.santi@unimore.it (D.S.); 2 Unit of Endocrinology, Department of Medical Specialties, Azienda Ospedaliero-Universitaria of Modena, 41126 Modena, Italy

**Keywords:** HIV infection, erectile dysfunction, libido, men who have sex with men, PDE-5 inhibitors, sexual health

## Abstract

Sexual issues tend to go unaddressed in human immunodeficiency virus (HIV) management, although overt sexual dysfunctions are more prevalent in people living with HIV than uninfected people. Erectile dysfunction is the most frequent sexual problem, with a prevalence of 30–50% even in men <40 years of age, but other issues such as loss of libido and ejaculatory disorders should not be overlooked. Peculiar factors related to HIV infection (e.g., fear of virus transmission, changes in body image, HIV-related comorbidities, HIV distress and stigma), alongside classical factors non-related to HIV, should be considered when approaching sexual problems in HIV patients. For this reason, the diagnostic and therapeutic workout of sexual dysfunction in the context of HIV requires a multidisciplinary approach, involving specialists in both infectious diseases and sexual medicine. This narrative review presents an overview of current knowledge on sexual dysfunction in HIV men, deepening the factors driving and taking part in these issues, providing advice for the clinical approach, and underlining the importance of caring for sexual health to improve the quality of life of HIV patients.

## 1. Introduction

A healthy and satisfactory sex life is considered an important element for a good health status and a gratifying quality of life [1,2]. Even though the relevance of a healthy sex life is a recognized issue also for people living with HIV [3,4,5], sexual problems tend to be unaddressed in HIV management [6]. Of note, different studies point out the higher sexual problems and overt sexual dysfunctions prevalence in people with HIV than in those who are HIV negative in both sexes [7,8]. Thus, people living with HIV continue to struggle with intimacy and physical pleasure because of several factors strictly linked to the HIV infection. Usually, sexual dysfunction and peculiar aspects of sexuality related to HIV remain in the background in the daily clinical practice with respect to the management of HIV infection itself and of major HIV-related comorbidities [6,9]. Overall, leaving sexual problems overlooked and undermanaged is known to further compromise the quality of life of these patients, which is already impaired by HIV distress, associated morbidities, and stigma [10].

In men, sexual dysfunctions, particularly erectile dysfunction (ED) [11], are more common among HIV-infected than HIV-uninfected men. These dysfunctions, especially if undermanaged, further impair the quality of life and general health, interfering with intimate relationships and lowering the adherence to antiretroviral medications [12,13,14], probably due to an individual’s belief that HIV medication itself may cause sexual dysfunctions. Thus, health professionals involved in the management of patients with HIV infection should address sexual difficulties not only to promote a healthy and satisfying sexual life, but also to enhance survival, improve general quality of life and relationships, and (through greater medication adherence) potentially nullify risk of transmission of HIV to another person [6].

## 2. Male Sexual Behavior in Men Living with Human Immunodeficiency Virus (HIV)

**Figure 1:**
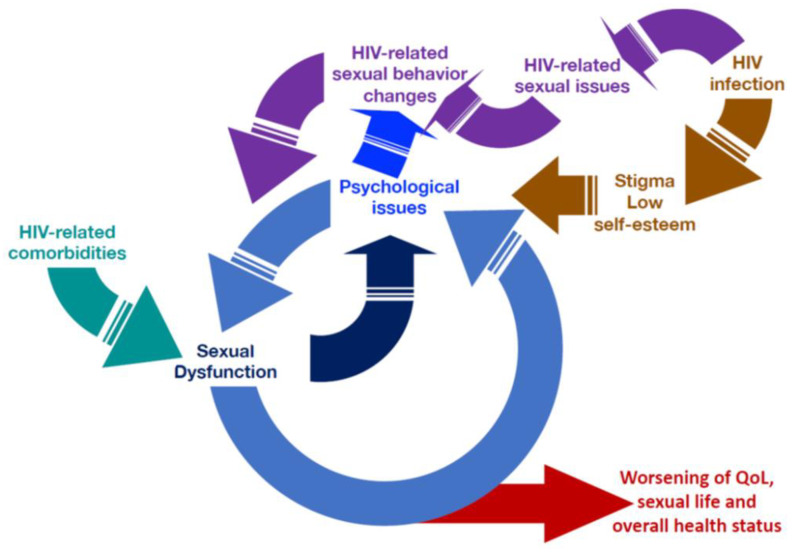
The vicious circle of sexual dysfunction, psychosexual issues, HIV infection and related morbidities and overall health status in men living with HIV. Abbreviations: QoL: Quality of Life.

When talking about sexual behavior in men with HIV several specific aspects should be taken into account (Figure 1, Table 1) [15].

In particular, ‘having’ the HIV infection may lead to changes in male sexual behavior or in psychological mood, indirectly affecting sexual behavior. Accordingly, the need to practice safe sex and the fear of HIV transmission may influence sexual behavior. Furthermore, the high prevalence of men who have sex with men (MSM) among men living with HIV compels considering peculiar aspects related to both sexual behavior (e.g., sexual practices) and overall sexuality (e.g., cultural and social factors) of gay men that may be relevant for the evaluation and management of sexual dysfunction [6,15]. Patients’ sexual preferences influence the type of sexual activity, getting involved in the modification of all phases of sexual activity from arousal to orgasm, especially in erectile function [6]. In addition, health problems associated to HIV infection may further impact on sexual behavior. Indeed, the sexuality of men living with HIV is multifaceted, involving psychological, social, cultural and pathophysiological factors, concurring in the development of sexual discomfort or overt sexual dysfunction; this results in a vicious circle that in turns impairs sexual function, quality of life, healthy sexual life and overall health status in men living with HIV (Figure 1). These peculiar factors are discussed below and are listed in Table 1.

## 3. HIV-Related Psychological Issues

The awareness of living with HIV poses several concerns regarding personal behavioral changes and how to relate with the partner. In particular, the fear of HIV transmission during sexual intercourse, how to disclose HIV status to the partner and its impact on sexual intimacy together with stigma are the main aspects involved in alterations of sexual behavior.

### 3.1. Fear of HIV Transmission

Fear of passing HIV to the partner during sexual activity is confirmed in about one third of MSM men and bisexual men with HIV infection [16]. Accordingly, men with HIV infection tend to have better scores of sexual function when masturbating than during sexual intercourses, suggesting that a safe practice, such as masturbation, interferes on sexual function less than risky sexual activities where the fear of HIV transmission is a relevant psychological concern [8]. The fear of HIV transmission impacts on all phases of sexual response, impairing satisfaction with sexual life [17], reducing sexual interest and promoting behaviors characterized by avoidance of sexual activity (i.e., sexual abstinence). These latter conducts could increase anxiety during sexual intercourse, potentially impairing both arousal, erection, and orgasm [17].

### 3.2. Disclosure of HIV Status to the Partner

Disclosure of HIV status to the partner could impact on patient’s approach to sexuality and on the relationship with the partner, due to the psychological implications that awareness of HIV status imposes/adds to the couple [18]. Disclosure of HIV status, in fact, is strictly related to stigma and depends on individual different approaches to the problem [19].

### 3.3. Stigma

Notwithstanding scientific and cultural educational efforts provided in the last decades for increasing awareness, HIV infection remains a stigmatized condition [20,21]. Stigma is highly prevalent in people living with HIV independent of gender, ethnicity and other factors [22]. Issues concerning gender identity and sexual orientation may further increase the stigma self-perception in MSM men [23,24,25].

Stigma related to men living with HIV and its direct and indirect effects on general health and sexual life have been a focus of research since the late 1980s. At a global level, stigmatizing attitudes towards non-heterosexual identities have long historical and religious origins in most countries that precede the HIV epidemic. The advent of HIV has reinforced discriminatory stereotypes already faced by many minority communities, such as gay people. Most literature on HIV-related stigma has been focused on three key areas: (i) perceived/felt stigma, which is the fear or belief that a person will be judged harshly or discriminated against; (ii) experienced/enacted stigma, which refers to acts of stigma or discrimination by others; (iii) internalized stigma, which is where individuals apply judgement or shame associated to stigma to themselves [26,27,28]. However, stigma can also manifest at social level, such as in discriminatory legislation and policies, acting as barriers for HIV men to access and maximize their use of health services [29,30].

Finally, body image changes related to HIV lipodystrophy contribute to increase self-perceived stigma especially in MSM [24], for which the body image preserves a central role [31,32]. Accordingly, some changes in the body shape involving also facial modifications are evident signs revealing the disease during social person-to-person interactions, enhancing the self-perceived stigma and low self-esteem in the social environment, including approach to sexual encounter.

Stigma plays also an important role in the pathogenesis of sexual dysfunction in HIV-infected men since it is highly related to psychological distress and low self-esteem, two conditions which may profoundly affect sexual behavior [6,33,34,35], especially in HIV-infected men [16,36].

### 3.4. Body Image

Body image may be impaired in men living with HIV as a consequence of body changes due to highly active antiretroviral therapy (HAART) induced lipodystrophy [37]. Moreover, from a cultural point of view, the role of body image and physical appearance is hampered in MSM community, which represents the vast majority of HIV-infected men [38]. Notably, the HIV men’s subjective perceptions of their physical appearance seems to have the major impact on their sexual well-being, rather than an objective changes in body itself [39]. It is likely that HIV-related stigma and discrimination exacerbate negative self-concept and self-image. Thus, the decreasing prevalence of objective changes in the body with HIV, especially lipodystrophy [37,40], will not result in a corresponding decrease in body image difficulties with HIV.

Ultimately, objective body changes and/or altered self-perceived body image are both associated to sexual dysfunction in HIV-infected men [41,42,43]. Besides, poor self-image is associated to loss of sexual desire in bisexual men and MSM [16]. Apart from the psychosexological correlates of body image perception, the metabolic alterations due to fat redistribution in the context of HIV-related lipodystrophy are known to more increase cardiovascular risk and impair cardiovascular health [44], involving also the penile district and ED [45].

## 4. Psychosexual Factors Related to MSM

### 4.1. Gay Culture

Men represent the majority of patients living with HIV in most countries. Among them the percentage of MSM is very high, up to 50% of all HIV-infected men in high income countries [46]. In these countries, the HIV epidemic is mainly driven by MSM [47], thus some peculiar aspects related to psychosexological and behavioral correlates of MSM need to be considered, influencing sexual function.

### 4.2. Importance of Body Image

Muscularity and body image are more important for gay and bisexual than heterosexual men, reflecting, at least in part, the place of honor that physical appearance and competition about the physical attractiveness occupy in gay subculture [38,48]. Thus, the great attention for physical attractiveness and muscularity could lead to body dissatisfaction and low self-esteem [42] and could prompt the abuse of anabolic steroids in MSM [49,50]. It is largely demonstrated that some of the latter (e.g., androgens) may interfere with sexual behavior [51], especially after their discontinuation [52].

### 4.3. Sexual Performance

Sexual performance is a relevant concern among MSM [53]. This high attention to sexual performance during intercourse, and sexual activity in general, inevitably leads to either ED, due to increased anxiety (i.e., situational anxiety due to performance failure), or premature ejaculation [53]. Moreover, a small reduction in erectile function (which usually occurs with advancing age) may be perceived as more severe even in presence of an erection that allows penetrative sexual intercourses. Accordingly, the need for better sexual performances is a recognized factor for promoting the use of drugs improving the erection for recreational purposes [54].

### 4.4. Anal Sex

The type of sexual intercourses in MSM may be of concern for erectile function. Indeed, the sexual activity practiced (i.e., anal penetration) may require a harder penile rigidity compared to vaginal penetration [55]. Sexual intercourses characterized by insertive anal sex may lead to self-perceived reduction of erection in the case of a small reduction of penile rigidity, prompting medical consultation for ED [56] or abuse of drugs for improving the erection [54].

### 4.5. Casual Sex and Group Sex

The attitude to casual sex and group sex among MSM accounts also for the need to guarantee a very good erection even in presence of a new partner (casual sex) and/or for a long period (group sex). In this context, yet a small erectile function impairment may be perceived as uncomfortable. Casual and group sex are affected by loss of intimacy, high fear of HIV transmission and a great attention to performance. All these issues increase anxiety and may impair sexual performance. Accordingly, both group and casual sex are strongly associated with the use of medications improving erectile function; the same occurs for insertive anal sex [56].

### 4.6. Recreational Drugs

The use/abuse of recreational drugs (e.g., anabolic steroids, alcohol, nitrate agents and psychoactive substances) to enhance and facilitate sexual intercourses is common among MSM [54], in both HIV-infected and non-infected people [57,58]. Some of these drugs may have undesired effects on sexual function [59], and they are included among ED risk factors [60,61]. Moreover, the use of recreational drugs is strongly associated to condomless, unsafe sex [62] and an increased risk of HIV transmission during sex. This remarks the importance of providing information on safe sex.

## 5. HIV-Related Sexual Behavior Issues

### Obligatory Condom Usage

Obligatory condom usage may exacerbate ED thanks to both objective and/or subjective (self-perceived) reduction of penile sensitivity and response to sexual stimulation during intercourses [63]. About 50% of MSM report to have experienced loss of erection using a condom [55,64]. In most cases, ED related to condom use is more frequent in men with lower erectile scores at International Index of Erectile Function (IIEF)-15 or experiencing ED even during intercourses without the condom [65]. This issue is of great concern for men living with HIV since condom slippage is common during intercourse for preventing the loss of erection, thus increasing the risk of HIV transmission [66]. For this reason, educational information on how to practice safe sex must be offered to all HIV-infected men seeking andrological consultation at any visit.

## 6. Arousal and Sexual Desire Dysfunction

About half of people living with HIV experience sexual difficulties [67,68], with loss of libido among the most commonly raised concerns [16,69,70]. Indeed, around 40% of HIV men who answered an anonymous self-filled questionnaire reported a general loss of interest in sex [67,71,72]. Despite the high prevalence of such sexual issues, reduced sexual interest and arousal are rarely investigated in men with HIV.

A complex network of multiple factors intertwined each other can be depicted at the basis of disturbances of sexual arousal in men with HIV. Low sexual desire (assessed by the proper IIEF-15 item) has been definitely associated with demographic factors (e.g., age, ethnicity) and HIV parameters (e.g., CD4 count, viral load, time since HIV diagnosis), and it occurs more frequently in men receiving HAART compared to HAART-naïve patients, especially those assuming protease inhibitors [13,67,69,73]. However, few studies with only small sample sizes have specifically investigated these aspects of medical treatment, leaving this association still inconclusive.

More variegated findings have been reported when non-medical factors were explored with particular regard to the emotional sphere. The association between men’s sexual dysfunction and psychological health is real and proven [74]. Depression and anxiety, two of the most important mental factors associated with sexual dysfunction [74], are quite prevalent in HIV series triggered by HIV distress and related emotional stressors [5,75,76]. In this way, an intricate system composed by psychological, social and relational factors experienced by HIV-infected men are linked to the reduced interest in sexual activities [72,73,77], in addition a poor health status may further contribute to lowering sexual desire [39,41,78].

As for other comorbidities commonly diagnosed in HIV-infected men, all abovementioned components causing poor sexual health can be subdivided into HIV-specific and classical risk factors. These latter are common to HIV-uninfected men, although probably potentiated in HIV context. For example, low sexual desire seems to be less dependent on circulating testosterone, given that a loss of libido was found in 65% of patients with normal serum testosterone levels [79], indicating that factors rather than androgens are a determinant of sexual desire in men with HIV.

## 7. Erectile Dysfunction in Men Living with HIV

### Prevalence of Erectile Dysfunction in HIV

ED is defined as a consistent or recurrent inability to achieve and maintain a penile erection sufficient to complete satisfactory sexual activity [80]. The reported global prevalence of ED in the general population is highly variable (from 3% to 76.5%) because of population selection, ED criteria, and modalities to evaluate erectile function [81]. It has been well described in the literature that the prevalence of ED is positively correlated with age: less than 10% in men under 40, 52% among men 40 to 70, and up to 70% in men over 70 years old [81,82].

Several studies reported a significantly greater prevalence of ED in HIV men compared to men without HIV [75,83], even after adjusting for age, body mass index, and risk categories [11]. It is worth noting the earlier ED onset in HIV-men compared to uninfected-HIV men. Indeed, ED is extremely common in middle-aged HIV-infected men [11], while it is uncommon in uninfected HIV-men before 50 years old [80].

Most of the studies analyzing the prevalence of sexual dysfunctions were conducted after the introduction of HAART. However, researchers evidenced issues of sexual dysfunction in this population also prior to HAART. Information about sexual life were collected mainly through self-administered questionnaires: difficulty in maintaining an erection, decreased libido and delayed ejaculation were the most frequent dysfunctions described in HIV men. Moreover, acquired immune deficiency syndrome (AIDS) patients were more likely to report sexual issues, suggesting that the greater the severity of the disease, the higher the prevalence of sexual dysfunction [84,85]. Increased prevalence of hypogonadism in these patients has been documented since the 1980s [86]. However, the real prevalence of sexual dysfunctions in that period has not yet been precisely determined, given the paucity of conducted studies and lack of validated instruments for the assessment of erectile function, such as IIEF-15, that was introduced only in 1995.

After the advent of HAART, with the improvement of patients’ general conditions and life expectancy, researchers devote more attention to the patients’ overall health, including their sex life. Accordingly, a large number of studies investigated the ED prevalence in HIV men following the introduction of HAART (Table 2). However, only a few works have provided clinical studies with control group of non-HIV populations. Overall, the ED prevalence in HIV ranges from 13% to 86% [11,45,69,73,75,79,87,88,89,90,91,92,93,94,95,96,97,98,99,100,101]. Studies conducted without the IEEF questionnaire [70,71,72,102,103,104] were excluded from the appraisal in order to expose more homogeneous data. However, a heterogeneous ED prevalence among populations is still observable, since sexual dysfunction is generally considered as secondary endpoint of the analysis.

As shown in Table 2, patients examined in the studies are middle-aged patients with HIV (age 38–54) who exhibit an increased prevalence of ED compared to middle-aged non-HIV-patients (Table 2). A recent meta-analysis [83] analyzed five case-control studies between HIV and non-HIV patients, confirming these findings. Patients affected by multiple chronic illnesses, particularly with cardiac and metabolic involvement, show higher ED rates as a different manifestation of a systemic vascular dysfunction [105]. In HIV men, the presence of baseline chronic illness and the HAART utilization, which disrupts the cardiovascular and metabolic systems, in conjunction with social stigma and the fear of transmitting the disease, amplify the risk of manifesting ED.

## 8. Pathogenesis of Erectile Dysfunction (ED) in Human Immunodeficiency Virus (HIV)

ED is a multifactorial condition that can be caused by an alteration of any of the components of erectile response, including psychogenic, endocrine, vascular and neurogenic comparts. Penile erection is triggered by erotic and emotional stimuli received or generated by the brain, especially by the limbic system (psychogenic erection) or by local sensory stimulation of genitalia mediated by peripheral nerve and sacral erection center of the spinal cord (reflex erection). The acquisition and maintaining of penile erection require an intact vascular system, which is facilitated by an adequate hormonal setting [105,106].

**Figure 2:**
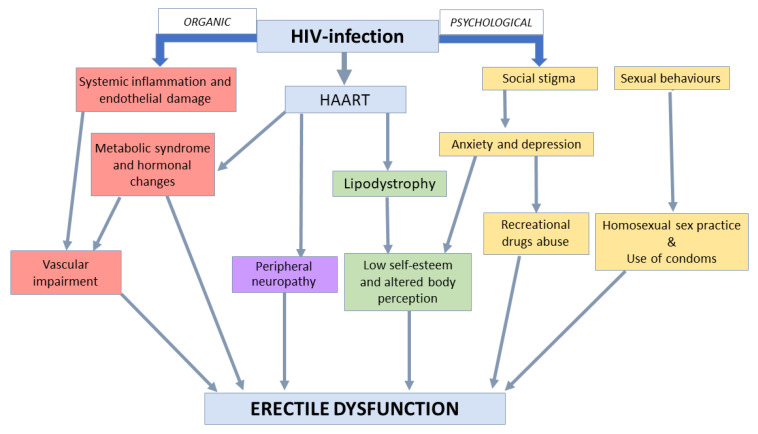
Organic and psychological factors involved in erectile dysfunction pathogenesis in men with HIV. Abbreviations: HAART: Highly Active Anti-Retroviral Therapy.

In clinical practice, ED is classically categorized on the basis of either organic—both endocrine and non-endocrine—or psychogenic etiology (Figure 2). Among organic, non-endocrine factors, the most common is vascular impairment, involving a deficit of arterial blood inflow or venous blood outflow (Figure 2). The presence of vascular arterial dysfunction could be an early indicator of a systemic vascular condition and often precedes major cardiovascular events [107]. HIV-infected men demonstrate increased risk for cardiovascular disease [108] as a result of the presence of classical risk factor for cardiovascular disease, such as metabolic syndrome, diabetes and obesity [109,110] and HIV-related factors, in particular systemic inflammation, immune system activation, and endothelial dysfunction [111]. However, a previous study [45] failed to demonstrate an association between vascular endothelial dysfunction and ED in HIV-infected patients, while another study found dyslipidemia as a main risk factor for ED in these patients [94]. Other disease involved in ED development, common in HIV-infected patients, is neurogenic impairment, such as peripheral neuropathy [112]. The association between peripheral neuropathy of the sacral region causing ED and the use of specific HAART drugs have been investigated, although with inconclusive results [103]. Moreover, the use of other drugs potentially inducing ED, such as antihypertensive and anti-depressant agents, are frequently used in HIV-positive patients [113]. In this setting, further risk factors for ED should be considered, such as cigarettes smoking [92] and the use of illicit and recreational drugs [114].

Endocrine factors are mainly related to testosterone deficiency, which occurs frequently in HIV-infected men [79,115]. Testosterone mediates a component of the penile erectile response through sexual desire, but studies in vitro have demonstrated that testosterone also plays a role in the cavernous smooth muscle, involving nitric oxide (NO), activity of RhoA-ROCK (Ras homolog gene family member A-Rho-associated) pathway and expression of phosphodiesterase type 5 (PDE-5) [116]. However, several studies fail to demonstrate a significant association between ED and the testosterone levels, both in HIV-infected [79,87,91] and HIV-uninfected men [81] and the percentage of men affected by ED remains elevated even in the presence of normal levels of circulating testosterone. The etiology of hypogonadism in HIV-infected men is multifactorial and hypotheses regarding the underlying mechanism have changed during the years. In the pre-HAART era, hypogonadism was mainly related to end-stage AIDS and poor general conditions, in conjunction with opportunistic infection [117]. In the HAART-era, hypogonadism with inappropriately low or normal LH levels (secondary hypogonadism) affects a conspicuous percentage of young to middle-aged patients and has been associated with altered body composition, virus-infection per se and HAART, and could be considered an overall expression of premature aging and patients’ increased frailty [79,118].

In HIV-infected men, other disease-related factors take part in the complex ED pathogenesis. Analogously to the general population, age constitutes one of the most important risk factors for ED. The process of ageing seems to be accelerated in HIV, having a significant impact on all the HIV-related comorbidities [45,69,119]. HAART has been widely investigated as possible direct cause of ED but the results are still inconclusive. Indeed, several confounding factors influence the analysis, such as the high number of different therapy regimens applied to treat HIV infection. Beyond these difficulties, HAART is also a main risk factor for other conditions that may indirectly cause ED, such as increased visceral adiposity, lipodystrophy, dyslipidemia and diabetes mellitus [120,121].

Among the anti-retroviral drug classes, protease inhibitors have been most widely studied in association with sexual dysfunction. Since the 1990s, several studies have found a link between ED and this class of drugs, although ED was investigated without using IEEF questionnaires [7,67,73,102,104], apart for two studies [91,97]. Other studies did not find an association between ED and use of protease inhibitors, using the IEEF questionnaire [69,88,89,99], or its adapted version [95]. A more recent study demonstrates that a protease inhibitor-containing regimen is a risk factor for ED in univariate models but not in multivariate models [97]. Unlike studies conducted in the 1990s and the first year of the 2000s, the most recent study evaluated protease inhibitor-drug regimen still currently used in clinical practice [97]. Ritonavir [67,102] and indinavir [102,104] have been reported to be associated with ED, while atazanavir has been associated with an improvement of sexual functioning [122]. In summary, sexual dysfunction and ED have been widely reported in association with protease inhibitor drug regimens, mainly in not controlled studies and with heterogeneous methods of sexual disorder evaluation. Nevertheless, when switching to an HAART regimen containing protease inhibitor, a sudden onset of ED is an important factor to consider in the diagnostic route.

The HAART therapy duration has been suggested to influence the ED onset in several studies [69,73,91,95], but not in others [45,75,87,90]. Considering the difficulties in distinguishing the latter as a risk factor from the duration of the disease itself and from the older age of the patients, the role HAART plays in sexual dysfunction—whether direct or indirect, negative or positive—remains controversial [83]. Past studies reported the CD4 count in association with ED [90], but other research fails to demonstrate the association [75,97,98].

Regarding other important factors involved in ED pathogenesis, lipodystrophy, consisting of to the redistribution of adipose tissue includes both fat accumulation (lipohypertrophy) and fat loss (lipoatrophy) or both [123], is frequently reported in HIV-infected patients to be related to ED [45,88,91]. Lipodystrophy is a direct consequence of the HAART-regimen and it radically impacts on quality of life and body perception, with profound effects on social life, sexuality and self-esteem [124]. Moreover, metabolic alterations linked to body fat redistribution represent a further risk factor for developing ED. Perhaps ED onset could be powered by the impact of HAART on body changes which could rise the psychological distress and anxiety [45].

Special attention should be spent for the psychological dimension, as depression and anxiety, extremely common in HIV-state [75]. These conditions represent one of the most important risk factors for ED in the general population and in HIV-positive men (Figure 2) [105]. States of depression are strongly associated with HIV-state [76] and, in conjunction with anxiety, contribute to decrease sexual response, facilitating the ED onset [90]. First, the perception of body image modification related to lipodystrophy increases sexual dysfunction both in HIV men and women [88]. Second, anxiety associated to the guilt and fear of transmitting the disease through sexual activity could have a huge impact on intimate relationships, and hence on erectile function [45]. Higher depression scores in HIV-infected patients were associated with sexual habits (MSM compared to heterosexual men), higher viral load and history of substance abuse [125]. Therefore, poor emotional status should always be contemplated in this setting of patients, since they present major risk factor in the development sexual dysfunction [97]. Depression seems to have a greater impact on sexual functioning compared to self-efficacy and stigma [39]. Thus, it is good practice to encourage patients to seek psychological counseling in order to stop this vicious circle.

In conclusion, HAART, premature aging and psychological factors appear relevant in the pathogenesis of ED in HIV-affected men. Moreover, factors associated with ED in normal populations seem to have less impact in this setting.

## 9. Clinical Approach to ED in Men with HIV

In HIV-infected men, not only factors traditionally related to ED must be considered, but the role played by the HIV infection per se [73], its associated comorbidities and the treatment [115] must considered. Hence, the clinical approach to sexual dysfunction in patients with HIV, both in terms of diagnosis and treatment, should consider either HIV-related or non-HIV related issues.

### 9.1. Diagnostic Work-Up

**Figure 3:**
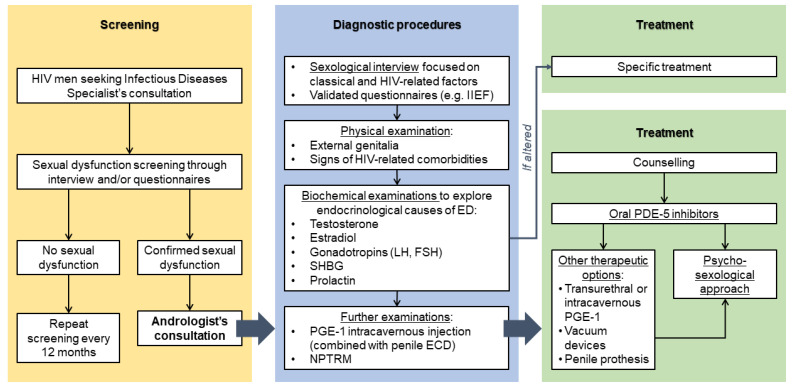
Diagnostic and subsequent therapeutic work-up to erectile dysfunction in HIV men. Abbreviations: IIEF, International Index of Erectile Function; ED, erectile dysfunction; LH, luteinizing hormone; FSH, follicle-stimulating hormone; SHBG, sex hormone-binding globulin; PGE-1, prostaglandin E1, ECD, eco-color-Doppler; NPTRM, nocturnal penile tumescence and rigidity monitoring; PDE-5, phosphodiesterase type 5.

As in the general male population, the diagnostic approach to sexual dysfunction in HIV patients should begin with an accurate interview aimed at collecting information on major contributing factors. Medical history should include psychosexological assessment as well as specific investigation of all above mentioned HIV-related factors concomitant drug therapies (i.e., anti-depressants, anti-psychotics, anabolic steroids, megestrol, lipid-lowering agents, etc.) and recreational drugs use (i.e., anabolic steroids, alcohol, nitrate agents and psychoactive substances, etc.) [126,127,128]. Special attention should be paid to data related to HIV infection per se and its implications, such as the presence of HIV-related comorbidities, body image and self-perception (dysmorphophobia), anxiety related to the chronic disease state and fear of virus transmission to the partner [41]. A detailed sexological interview is recommended to objectify and quantify the patient’s complaint [126,127,128] (Figure 3). During the interview the physician should investigate in depth all aspects of the sexual sphere, without being reticent to discuss such issues with the patient [3,14]. All the specific HIV-related issues listed in Table 1 should be investigated asking to the patient if they are relevant or not for him. Physicians must not be reluctant to investigate patients’ sexual life, even though it has been considered a taboo in the past. The most recent European Guidelines on HIV define sexual dysfunction as a comorbidity that disproportionately affects HIV men. Thus, sexual health must be routinely assessed in clinical practice alongside other system disorders during HIV consultation [4].

In particular, the interview must include a proper framework aimed at exploring sexual orientation, partner’s serological status, and sexual activities practiced (oral, anal, vaginal intercourses) to better contextualize and manage patient’s sexual dysfunction. Furthermore, the moment of the interview should be seen as an occasion to reinforce counselling on topics of ethical concern, including protective behaviors on HIV transmission to the partner in serodiscordant couples [23,129] and investigation of the most common factors inducing the patient to practice condom slippage during intercourses. In this way, the interview acquires a double value, being a diagnostic tool with important educational and therapeutic potential. A well-conducted interview avoids underdiagnosis and, consequently, undermanagement of ED. Notably, all HIV individuals under regular follow-up should have a sexual health assessment at first presentation and at 12-monthly intervals thereafter, even for those patients not presenting any sexual dysfunction at the first evaluation [3] (Figure 3).

At this stage, validated questionnaires should be submitted to the patient for ruling out ED (Figure 3). The IIEF is the most used tool [130] and it is available in two forms, IIEF-15 and IIEF-5, composed by 15 and 5 items, respectively [131,132]. However, since the IIEF-15 was originally created as a diagnostic tool aimed at heterosexual men, it might be not adequate for MSM, who represent the majority in HIV cohorts. Furthermore, the IIEF-15 items do not explore sexual habits, such as anal intercourse, which are common among MSM. For these reasons, an adapted IIEF-15 questionnaire investigating also sexual behaviors engaged by MSM has been developed and validated for MSM [55,66,133]. To date, only the English version of this modified questionnaire is available [133]. Beyond the IIEF, other validated tools for ED diagnostic purposes could be applied (e.g., Structured Interview of Erectile Dysfunction, SIEDY [134]), presenting the same reliability of IIEF, the questionnaire to be used being chosen according to physician’s preference and experience independently from his/her expertise in sexual medicine. By contrast, the role of the infectivologist is to collect detailed information on patient’s sexual health in order to select those who need management by other consultants such as an andrologist, endocrinologist, urologist, sexologist [135,136].

Once the presence of ED is confirmed by interview and/or questionnaire outcomes, the diagnostic work-up requires further physical and biochemical examinations aiming at ruling in/out any metabolic and endocrine disease, such as hypogonadism, dyslipidemia, and diabetes mellitus [6,79,88,137] (Figure 3). Among these, hypogonadism is one of the most frequent HIV-related endocrine comorbidities in men [79,115] and its diagnosis in HIV could be tricky [115]. Classical signs and symptoms of hypogonadism (e.g., loss of vitality, reduced sexual desire, loss of muscle mass) should be carefully investigated to detect a possible condition of testosterone deficiency. An accurate exploration of external genitalia should evaluate testicular size and consistency, and exclude signs of sexually transmitted diseases, whose incidence is higher in HIV setting [3,70]. Since clinical features of low serum testosterone in HIV-infected men are aspecific, of mild-to-moderate degree and often overlapping with those of HIV infection per se, its clinical detection can be underestimated, and only laboratory blood examinations allow identifying the presence of biochemical hypogonadism. Thus, biochemical examinations of the hypothalamic-pituitary-gonadal axis are recommended for all HIV-infected men, especially those presenting sexual dysfunction. Serum total testosterone circulates mainly bound to sex hormone binding globulin (SHBG) and albumin, but only the small fraction of non-protein bond or free testosterone is responsible for the biological activity of T [138,139,140]. Abnormalities in SHBG levels can influence the total testosterone serum levels. In particular, increased SHBG serum levels are typically detected in HIV-infected men, leading to normal total testosterone serum levels in spite of actually low free testosterone levels, causing biochemical hypogonadism [115]. Thus, the mere measurement of total testosterone could mask a condition of biochemical hypogonadism and the assay of SHBG for the calculation of free testosterone levels is required for the correct evaluation of the gonadal status in HIV men [115].

Other examinations, such as dynamic penile color duplex ultrasonography or intracavernous drug injection (ICI), might be useful to discriminate organic from psychogenic forms of the disease [126,127] (Figure 3). Prostaglandin-E1, whose synthetic analogue is alprostadil, is the vasoactive agent most frequently used for the pharmacological induction of penile erection [126,141]. The occurrence of a normal erection within 10–20 min after ICI is suggestive for a normal veno-occlusive mechanism [141]. By contrast, the lack of erection or a partial erection after prostaglandin-E1 ICI are diagnostic for an organic vascular component of ED, without distinguishing between a venous or an arterial origin of the vascular disease [126,127]. Starting from the minimum, the prostaglandin-E1 dose has to be progressively increased to find the dose required to induce a complete erection. This information has, at the same time, a diagnostic and a therapeutic meaning since it allows us to quantify the severity of vascular impairment and it can be proposed as adequate dose for intracavernous self-injection therapy [126,127]. The ICI examination could be completed by the use of dynamic penile color-Doppler ultrasonography that performs a dynamic evaluation of blood flow during the erection, adding important information especially about penile arteries rather than veins [126,127]. In particular, a systolic (>25 cm/s) and/or diastolic (<5 cm/s) peak of flow velocity can rule out arteriogenic and venogenic ED, respectively [126,127]. Considering other less used investigations, nocturnal penile tumescence and rigidity monitoring might be useful to confirm the psychogenic cause of ED when normal nocturnal erections are clearly documented [126,127].

A multidisciplinary approach is suggested to optimize the ED diagnostic and therapeutic work-up in HIV patients. Specifically, the infectious diseases specialist should manage the patient’s general health condition, the infection stage and evolution, and antiretroviral drugs. The endocrinologist should evaluate and control the coexistence of endocrine, andrological and/or metabolic disorders, which may concur in the ED onset. A psychologist/psychiatric intervention is fundamental to diagnose and control any mood disturbance, such as depression, or to manage a recognized psychological component of ED. Finally, all specialists should promote in concert safe sexual practices, aiming at reducing the overall risk of HIV transmission. It is important to ensure the patient understands the possibility of virus transmission and its implications, underlining that an undetectable viral load does not nullify the possibility of infecting the partner, suppling information on safe sex and not promoting unprotected sexual intercourses [3,55].

### 9.2. Erectile Dysfunction (ED) Treatment in HIV

ED treatment is mainly based on a pharmacological and psycho-sexological intervention for men with HIV, as well as for uninfected men [3,127] (Figure 3). Anyhow, the impact of all modifiable risk factors that might affect the erectile function (e.g., substances abuse, overweight, sedentary lifestyle, cigarette smoking) should be reduced by providing adequate counselling [127].

### 9.3. Pharmacological Treatment

Before starting any specific treatment for ED, the infectious disease specialist should consider modifying antiretroviral drugs, according to the patient’s virological status-related parameters (i.e., viral load, CD4 count). The regimen change could be beneficial especially for those patients who complain of the onset of ED soon after starting the drug. Even though a placebo effect of this strategy is reasonable and could not be excluded a priori, this remains a valid and successful approach to be applied, when possible [67].

Considering specific pharmacological treatment, orally PDE-5 inhibitors are the first-line agents used to manage ED. Four PDE-5 inhibitors are currently available: sildenafil, tadalafil, vardenafil, and avanafil. The safety profile of PDE-5 inhibitors for HIV men is the same of uninfected men [3]. However, interferences between antiretroviral therapy, especially protease inhibitors, and the metabolism of PDE-5 inhibitors have been described [3,142,143]. The lowest starting dose is recommended and titrated according to response and side effects. Patients who use nitrates and nitrate-containing compounds, including recreational use of inhaled amyl nitrate, should be cautioned not to use these agents in conjunction with PDE-5 inhibitors. Because both PDE-5 inhibitors and nitrates are vasodilators, coadministration can have synergistic effects triggering marked vasodilation and severe hypotension through excessive accumulation of cyclic guanosine monophosphate (cGMP) [144].

The use of PDE-5 inhibitors in a HIV setting implies some ethical concerns about the possible promotion of virus transmission [145]. No risk behavior increase using PDE-5 inhibitor has been detected [146,147], but the use of these medications in conjunction with other risk-related substances (drugs and alcohol) tends to worsen a risk-taking profile triggering HIV-related risk behavior. In this sense, a good patient-physician relationship and an accurate interview are encouraged to identify risky sexual practices, patient’s expectations, or other medical factors that might discourage the use of PDE-5 inhibitors.

A real thorny issue is represented by the misuse of PDE-5 inhibitors as recreational drugs without medical consultation [145,148,149]. Access to PDE-5 inhibitors s by non-conventional methods (Internet prescribing) does not normally allow for a proper discussion on safer sex, or discussion around safe use of these drugs with recreational agents. Without a previous medical consultation, physician has not the possibility of stemming risky sexual practices boosted by experiencing a complete erection, albeit pharmacologically induced, and further fomented by the possible concomitant assumption of other legal and/or illicit substances (e.g., drugs, alcohol) [145,148,149]. Unprotected sex without a condom, promiscuity including group sex and sex workers, who use PDE-5 inhibitors to guarantee sexual performance, are examples of unsafe behaviors. This modality of PDE-5 inhibitors misuse lies outside its adequate use in an appropriate clinical context. Men who use PDE-5 inhibitors regardless of medical opinion generally present other psychosexological disturbances, such as compulsive sexual behaviors and an ideal of sexual performance to be reached above its normality [145]. The conduct of patients who undergo proper diagnostic investigations for ED is different. In this case, once the ED diagnosis is made, patients receive regular prescriptions for drugs, beyond fundamental information on safe sex and counselling. In conclusion, even though ethical concerns about the prescription of PDE-5 inhibitors in HIV have been raised [150], any physician should act without judgement to minimize the well-known social stigma, reserving to HIV patients the same right as other individuals to have a satisfying sexual life [23].

Before prescribing PDE-5 inhibitors, the presence of hormonal disorders, which require specific treatment, should be ruled out. For example, ED in the context of documented hyperprolactinemia or hypogonadism is expected to improve, even until complete restoration, after starting therapy with dopamine agonist or exogenous testosterone, respectively. In hypogonadal HIV patients, testosterone replacement therapy generally helps in raising sexual desire and erection quality [127], enhancing PDE-5 inhibitors’ effectiveness [151].

For men who have contraindications to the use of oral PDE-5 inhibitors or find them ineffective, other second-line medical or surgical treatments should be considered, including the prostaglandin-E1 ICI or transurethral application [126,127]. Men should be thoroughly informed regarding the benefits and potential risks. The most serious, albeit rare (mean 1.8%) [126], adverse event associated with ICI is priapism. Moreover, pain, penile fibrosis or plaque and penile deformities have been reported with various incidence (4.5–13%) after chronic ICI therapeutic application [126]. Furthermore, it has to be noticed that there is the possibility of virus transmission whether HIV-uninfected people are accidentally injured with the infected needle. Hence, once ICI should be prescribed, the patient must receive an in-office injection test and he must perform regular self-monitoring of the penile structures, in order to early detect the appearance of increased-consistency areas suggestive for fibrosis [126]. Once the presence of fibrosis is documented by palpation and ultrasonography, the andrologist may consider interrupting ICI therapy, since the fibrosis could progress, causing a curvature of the penis that does not allow sexual intercourse in the most severe cases. Additional counselling on safe sex by using a condom even to completely cover the injection site should also be provided.

In cases where all medical therapies have failed, the choice of vacuum device or the surgical option of penile prosthesis implantation remain the only therapeutic choices to be considered [126]. Given the invasive and irreversible nature of penile prosthesis surgery, men and their partners should be thoroughly counseled regarding the benefits and potential burdens of this treatment to ensure appropriate choice of device, realistic post-operative expectations, and potential for high satisfaction [126].

Psychological and psychosexual issues may not to be underestimated as contributors to ED, especially in HIV setting [14,70,72,152]. Thoughtful discussion of these issues with men and their partners is a key component of patient education and can promote acceptance of incorporating a mental health/sexuality expert into the therapeutic plan. Psychotherapy and psychosexual counseling focus on helping patients, improve communication about sexual concerns, reducing anxiety related to entering and during a sexual situation, and introducing strategies for integrating ED treatments into their sexual relation. For HIV men with predominantly psychogenic ED, the use of PDE-5 inhibitors for a short period might reduce the anxiety related to erectile failure. Hence, these patients should be referred to a psychotherapist as either an alternative or combined to medical treatment to ED [41,88,136]. Psychosexological counselling could be provided even in cases of organic ED to reduce the associated psychological component, aiming at limiting the perception of stigma related to HIV infection, reducing anxiety caused by sexual dysfunction itself and, finally, including advice on safe sex. Overall, the risk of HIV transmission to the sexual partner inevitably influences sexual behavior. It happens that some patients might be reluctant to assume medications for ED to not incite sexual activity because of fear of HIV transmission [55]. Especially for such patients, the involvement of a psychologist in the multidisciplinary team is fundamental to offer proper counseling.

## 10. Disorders of Ejaculation

Ejaculation dysfunction can be classified as premature, delayed/retarded or retrograde. Retrograde ejaculation is characterized by the reflux of seminal fluid within the bladder, caused by the failure of the bladder neck to close [153], whereas, delayed ejaculation is the persistent difficult to ejaculate without alteration of sexual desire and erection [154]. Premature ejaculation (PE) represents the most frequent reason for andrological consultation in men and could be further divided into primary, whether it occurs since the first sexual intercourse, or secondary, if it arises several years after sexual activity initiation. Many risk factors and different potential pathogenesis have been identified with regard to the secondary form, such as psycho-relational factors, sexual dysfunction, and endocrine, urologic or idiopathic factors [155]. Hence, in HIV-infected men, many factors may concur in affecting ejaculatory function.

Prevalence of ejaculation dysfunction in HIV has not yet been determined, since very few studies have been conducted and they focus almost entirely on a specific population of HIV-infected MSM. Generally, the prevalence of ejaculation disorder in HIV is reported to be around 39% (36–42%) [67]. This hypothetical prevalence rate has been obtained using a large variety of heterogeneous diagnostic tools, ranging from validated, self-administered questionnaires, such as the Premature Ejaculation Diagnostic Tool (PEDT) [156] or the Premature Ejaculation Profile (PEP), to simple questions on the topic during clinical interview. Thus, data on ejaculation disorder in HIV remains controversial. Hirshfield et al. investigated the presence of sexual dysfunction in MSM through self-administered questionnaires online, detecting that HIV-infected men reported all of the symptoms of sexual dysfunction more frequently than non-HIV patients, with the exception of PE [157]. These data are in line with the results of another small observational study [158], while another self-reported survey in MSM-HIV reported that 31% of them suffered from PE, 24% from retarded ejaculation and 19% from anorgasmia [71]. However, a study with a control population of non-HIV patients failed to demonstrate an association between PE and HIV status [61]. The incidence of ejaculation disorders in HIV-infected men seems unrelated to either the HIV infection duration or the patients’ age, although HIV+ status is considered one of the main risk factors for developing PE in MSM, together with the presence of low urinary tract symptoms (LUTS), sex life dissatisfaction, the lack of a steady relationship, few sexual partners and a lower level of education [61,93,159].

Conversely, delayed ejaculation in HIV-infected men has been associated with peripheral neuropathy, which disrupts the autonomic sensory process of ejaculation, and with use of antidepressant drugs [60]. An association between didanosine and ejaculatory disturbance has been reported, but scientific evidence of HAART-induced sexual dysfunction remains unclear, as previously discussed [67,160].

In conclusion, robust evidence of an association between HIV-infection and risk of ejaculation disturbance is so far unavailable. In order to clarify existing evidence, more studies are required, and should include also heterosexual patients and a control HIV-uninfected population.

## 11. Impact of Fertility Concern, and Diseases of the Seminal Tract on Sexual Dysfunction in HIV

Sexual dysfunctions could be the consequence of psychological distress and emotional worries, among which, fertility-related issues could play an important role [161]. The improvement in HIV-related treatment led to an increased life expectancy together with a lowered risk of HIV transmission, both resulting in boosting the desire of HIV-infected people to have children [162,163]. However, when an HIV-infected patient, both male and female, desires a child, many concerns causing important psychological stress must be considered. From the male point of view, this psychological distress, could finally lead to ED or, more generally, to sexual dysfunction. An HIV-infected man who wants to have a child is aware about certain specific risks, such as the possibility of vertical and horizontal viral transmission. Indeed, HIV is detectable in the semen, both shortly after primary infection [164], and subsequently in all other stages of infection [165]. Moreover, the seminal viral load changes according to the disease stage and the efficacy of antiretroviral therapy applied [166,167]. With this in mind, the semen remains the main vector of HIV transmission, carrying three specific viral vectors: free virions, spermatozoa-associated virions and infected leukocytes [167,168]. This is obviously a relevant problem in terms of viral transmission, but also a potential trigger of male infertility. Indeed, the HIV presence in the male seminal tract triggers the production of several cytokines and chemokines, such as interleukin (IL)-1b, IL4, IL6, IL7, IL8, granulocyte macrophage-colony stimulating factor (GMCSF) and monocyte chemoattractant protein (MCP)1 [169,170]. This inflammatory storm shows a double negative effect, sustaining the viral replication [170], and creating the immune environment unfavorable to normal sperm production [170]. Accordingly, the role of inflammation on spermatogenesis alteration is largely demonstrated in the general population [171,172,173] and in many sexually transmitted diseases (STDs) [174], such as HIV infection, in which a low sperm concentration, an high percentage of sperm with abnormal morphologies and an high percentages of DNA damage were widely demonstrated [175,176,177]. Moreover, a progressive sperm quality impairment is described with the progression of the HIV-related disease [177,178]. Thus, it is now clear that HIV-infected men could be infertile or sub-fertile, as justified by the increasing number of HIV-infected people accessing the assisted reproductive centers (ART), which is a safe option once the semen has been washed free of HIV [179,180]. With all these aspects in mind, an HIV-infected man could have infertility concerns, related to a sperm quality impairment, which, combine with the already described psychological issues (Figure 1).

The problems and doubts related to fertility are much more complex and must also consider the social environment in which the patient lives. There is large literature highlighting that reproductive desires of HIV-infected subjects are not perceived as significantly different from those who are not infected, although the majority of these studies are mainly focused on the female partner [181]. However, people living with HIV are still forced to face stigma related to their infectious disease when they try to exercise their reproductive rights. In particular, several studies suggested that HIV-infected men and women often fear judgement from their healthcare providers because of their fertility desires [182,183]. Moreover, in several countries worldwide a negative attitude about childbearing is still evident among people living with HIV, encouraging to cease childbearing [184,185,186]. This was particularly true in the pre-HAART era, when there was an attitude to stigmatize childbearing [187]. In recent years, reproductive technologies have been improved and the risk of HIV transmission during pregnancy has been drastically reduced [188]. These policies support the fundamental right of HIV-infected men and women to approach reproductive health services [188]. However, although appropriate interventions, such as HAART adherence and ART approaches [179,180], could limit the risk of viral transmission, the fear of unsafe conception could reinforce the original psychological stress to HIV-infected men. In general, no studies have so far evaluated sexual dysfunction consequent to HIV-related infertility concerns. There are a few studies in the literature that have evaluated the perceptions and the psychological consequences of sexually transmitted infections in young men, however they are limited to contraception and not to childbearing [189].

Finally, the sexual dysfunction in HIV-infected men could be further enhanced by STDs recurrences, which are also directly related to infertility and subfertility [174,190]. Indeed, co-infection with multiple pathogens is common worldwide in people living with HIV [191,192,193,194,195]. Among these, several SDTs could lead to physical consequences, both anatomical, hormonal and neurological, which can further aggravate sexual dysfunction. Syphilis, for example, is increasingly detected as an HIV-co-infection [196,197] and it could lead to neurological disturbances that progressively impair erectile function. Moreover, the presence of LUTS caused by STDs in HIV-infected men could further increase the sexual dysfunction [198,199]. This was widely demonstrated in the general population, but it is also true in HIV-infected men, in which the highest LUTS presence is associated with the poorest sexual function [159]. However, only a limited number of trials have evaluated the sexual function of HIV-infected men in relation to their co-infection. Since HIV-infected men are more susceptible to genital tract infections and given that these conditions could lead to sexual dysfunction, it is logical to assume that men with HIV have an increased risk of sexual dysfunction. Nonetheless, specific epidemiological and cross-sectional trials should be designed to discriminate how these aspects could together trigger sexual dysfunction.

## 12. Conclusions

Sexual dysfunctions are common among HIV-infected men. Several HIV-related factors and sexual behavior habits lead to more concerns about sexuality in MSM with HIV in terms of both prevalence of sexual dysfunctions and self-perceived impairment of sexual function. Among all the clinical issues to be investigated and monitored in HIV-infected men (from the infection itself to all the other comorbidities and quality of life) sexual function and dysfunction risk remaining in the background. For this reason, physicians who manage HIV-infected men must be aware of sexual problems in HIV and should include questions useful to know more about the patient’s sexual life and the relative degree of satisfaction. The educational moment devoted to how to practice safe sex may be a good starting point to investigate a patient’s sexuality and sexual behavior during a clinical interview and to point the patient to an adequate work-up or to an andrological consultation. Apart from the classical work-up useful for the diagnosis and management of male sexual dysfunction, several peculiar issues related to HIV infection and MSM psychological and behavioral pattern must be considered since they may influence sexual behavior and sexual performance.

## Figures and Tables

**Table 1:** Specific factors that influence sexuality in human immunodeficiency virus (HIV)-infected men.

**HIV-Related Organic Factors**
Effect of HIV-related comorbidities (e.g., hypertension, diabetes mellitus, dyslipidemia) on cardiovascular function
**HIV-Related Psychosexological Issues**
Fear of virus transmission
Disclosure of HIV status to the partner
Stigma
Body image changes (i.e., lipodystrophy)
**MSM Related Psychosexological Issues**
Gay Culture
Self-perceived body image
Body image importance in gay culture
Importance of sexual performance
Anal sex
Casual sex and group sex
Recreational drugs abuse
**HIV-Related Sexual Behavioral Issues**
Obligatory condom use

**Table 2:** Studies that investigated erectile dysfunction (ED) and reduced libido prevalence in HIV patients.

Study	*n*	Age	MSM (%)	IV Drug Use (%)	Reduced SD (%)	ED (%)	ED Diagnostic Tool
Lallemand et al. (2002) [89]	156	40.5 ± 7.7 *	100	NR	89	86	IIEF-15
Ende et al. (2006) [87]	118	41 (28–67) §	70	7	NA	74	IIEF-5
Asboe et al. (2007) [69]	668	-	73	-	24	33	IIEF-15
Crum-Cianflone et al. (2007) [90]	285	39 (19–72) *	NR	8	NR	61.4	IIEF-15
Guaraldi et al. (2007) [88]	357	45 (45–46) *	43	NR	NR	53.2	IIEF-15
Moreno-Pérez et al. (2010) [91]	90	42 ± 8.2 *	80	0	NR	53.4	IIEF-15
Rochira et al. (2011) [79]	247	45 (20–69) §	NR	NR	65.2	53.4	IIEF-15
Guaraldi et al. (2012) [45]	133	49 *	52	26	NR	59.3	IIEF-15
Zona et al. (2012) ¶ [11]	444	44.8 ± 5.9 *	43	34	NR	54.5	IIEF-15
Hart et al. (2012) ¶ [92]	1340	48 (42–54) §	100	NR	NR	21	IIEF-MSM
Vansintejan et al. (2013) [93]	72	41 ± 10 *	100	NR	15	56	IIEF-5 **
Wang et al. (2013) [95]	4064	46 §	NR	NR	NR	24	IIIEF-15 ‡‡
Perez et al. (2013) [75]	158	46 *	58.2	NR	NA	67.1	IIEF-5
Romero-Velez et al. (2014) [94]	109	39.9 ± 8.8 *	70.6	NR	NR	65	IIEF-15
Hart et al. (2015) ¶ [101]	619	47.3 ± 8.9 *	100	NR	NR	24.7	IIEF-MSM
Pinzone et al. (2015) [96]	109	47 (40–52) §	54	20.2	NR	65	IIEF-15
Fumaz et al. (2017) [97]	501	42 (35–48) §	75.8	NR	NA	58.4	IIEF-5
Aghahowa et al. (2017) [98]	217	37.9 ± 9.9 *	63.6	9.5	NR	82.3	IIEF-15
Dijkstra et al. (2018) ¶ [73]	399	53.6 (48.6–60.0) §	100	2	7	13	IIEF-15 ‡‡
Veras Gomes et al. (2019) [99]	134	44.7 ± 11.0 *	40	NR	NR	22	IIEF-15
Bernal et al. (2019) [100]	139	45.22 ± 10.47 *	55.6	3.7	NA	61.2	IIEF-5

* Mean ± standard deviation § Median ± interquartile range All studies were cross-sectional, non-controlled except for ¶ prospective controlled study. ** Web-based self-filled International Index of Erectile Function-5 (IIEF-5). ‡‡ In these studies, the authors used a not-validated modified version of IIEF-15. Abbreviations: IIEF, International Index of Erectile Function; ED, erectile dysfunction; HAART, highly active antiretroviral therapy; IV, intravenous; MSM, men who have sex with men; NA, not applicable; NR, not reported; SD, sexual desire.
